# A validated high performance liquid chromatography method for the analysis of thymol and carvacrol in *Thymus vulgaris* L. volatile oil

**DOI:** 10.4103/0973-1296.66927

**Published:** 2010

**Authors:** H. Hajimehdipoor, M. Shekarchi, M. Khanavi, N. Adib, M. Amri

**Affiliations:** Traditional Medicine and Materia Medica Research Center and Department of Traditional Pharmacy, School of Traditional Medicine, Shahid Beheshti University of Medical Sciences, Tehran, Iran; 1 Department of Research and Development, Food and Drug Control Laboratories and Food and Drug Laboratory Research Center, MOH & ME, Tehran, Iran; 2 Department of Pharmacognosy, Faculty of Pharmacy, Tehran University of Medical Sciences, Tehran, Iran; 3 Department of Pharmacognosy, Pharmaceutical Sciences Branch, Islamic Azad University, Tehran, Iran

**Keywords:** Carvacrol, gas chromatography, high performance liquid chromatography, thymol, *Thymus vulgaris*, validation

## Abstract

*Thymus vulgaris* L. (Lamiaceae) is a well-known medicinal plant that contains important compounds such as thymol and carvacrol and it has been used in many pharmaceutical dosage forms. Thymol and carvacrol in essential oils are often quantified by gas chromatography (GC) technique but in this work, a validated and reliable high performance liquid chromatography (HPLC) method has been developed for the analysis of these two components in *T. vulgaris* essential oil. The essential oil of the plant was analyzed by HPLC and GC techniques. The HPLC system consisted of ACE C_18_ column and an isocratic acetonitrile:water (50:50) as the mobile phase which was kept at a flow rate of 1 ml/min. The method was validated for selectivity, linearity (*r*^2^ > 0.997 for both thymol and carvacrol), precision (intra-day 0.8-1.9, 1.7-2.6; and inter-day 3.5-4.5, 3.6-4.7) and recovery (97.7%, 97.6%) for thymol and carvacrol, respectively. The limits of detection (LODs) and limits of quantization (LOQs) were calculated to be 2.8, 0.6 µg/ml and 8.6, 1.8 µg/ml for thymol and carvacrol, respectively. The GC system consisted of flame ionization detector (FID) and CP-SIL 8 column. The concentrations of thymol and carvacrol in essential oil obtained by HPLC (41.2%, 4.3%) and GC (40.7%, 4.2%) were compared by statistical methods and they showed good agreement.

## INTRODUCTION

**Figure 1:**
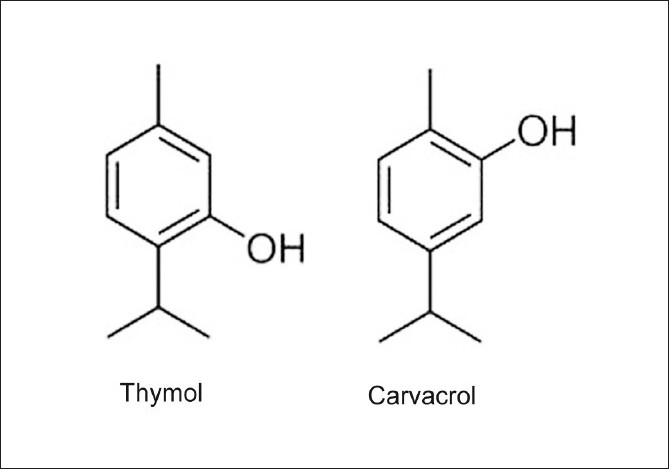
Structures of thymol and carvacrol

The genus of *Thymus* comprises 300–400 species, some of which are used in folk medicine. The main medicinal *Thymus* is *Thymus vulgaris* (common thyme) which is used for dry coughs, bronchitis, laryngitis, indigestion and gastritis.[1,2] There are also some reports about antimicrobial effects of *Thymus* essential oil.[3] It has been demonstrated that the biological effects of *T. vulgaris* are mainly due to the presence of phenolic compounds, especially thymol and carvacrol [Figure 1].[2,4] Thymol content in thyme essential oil is much higher than carvacrol content. This compound shows 30 times higher antiseptic effect and four times lower toxicity than phenol.[5,6] Since these two phenolic components of the plant are considered as active constituents, most of the pharmaceutical dosage forms are standardized according to their thymol or carvacrol contents. Therefore, introducing a validated method for the assay of these compounds should be considered. Gas chromatography (GC) is the most popular method for analysis of herbal volatile components,[1,7–9] but it is not possible to determine thymol and carvacrol in pharmaceutical dosage forms directly. Therefore, usage of other methods in quantization of volatile compounds in complicated matrixes is obligated.[10–12] High performance liquid chromatography (HPLC) is one of the most precise techniques for quantitative determination of plant constituents, which is used for volatile and non-volatile compounds.[13,14] In this research, a validated HPLC method for the assay of thymol and carvacrol in *T. vulgaris* volatile oil has been introduced and concentrations of these two phenolic compounds in the essential oil which were measured by the new HPLC technique compared with those obtained by GC method.

## MATERIALS AND METHODS

### Plant material

Aerial parts of *T. vulgaris* were collected in July 2008 from Gorgan (Golestan province) and identified by M. Khatamsaz, Botanist, Research Institute of Forests and Rangelands, Tehran, Iran.

### Isolation of essential oil

The air-dried and powdered aerial parts of the plant were subjected to hydro-distillation for 4 h using a Clevenger type apparatus. The obtained essential oil was dried with anhydrous sodium sulfate and stored at +4°C before using.

### Chemicals

Thymol and carvacrol standard materials were purchased from ROTH (Karlsruhe, Germany). All the solvents prepared were from Merck (Darmstadt, Germany).

### Instrumentation

HPLC experiment was performed using a Waters Alliance system equipped with a vacuum degasser, quaternary solvent mixing, auto-sampler and a waters 2996 diode array detector. UV spectra were collected across the range of 200–900 nm, extracting 274 nm for chromatograms. Empower software was utilized for instrument control, data collection and data processing. The column used was an ACE C_18_ (4.6 × 250 mm, 5 µm). The mobile phase was an isocratic combination of acetonitrile (ACN):H_2_O (50:50) with a flow rate of 1 ml/min. Injection volume for all samples and standard solutions was 10 µl.

GC was carried out using a Varian CP-3800 GC with a capillary column CP-SIL8 (60 m × 0.25 mm i.d., 0.25 µm f.t.). The carrier gas used was N_2_ at a flow rate of 1 ml/min and a split ratio 1:30. A flame ionization detector (FID) was used. The column temperature was programmed at 50°C for 1 min and then heated to 265°C at a rate of 2.5°C/min; injector temperature was 260°C, detector temperature was 300°C, H_2_ flow was 30 ml/min and air flow was 300 ml/min.

## High performance liquid chromatographic quantization of thymol and carvacrol

### Sample preparation

Ten milligrams of essential oil was added to a volumetric flask and diluted to 100 ml with ACN:H_2_O (80:20) (100 µg/ml). Six samples were prepared and each one was injected three times.

### Preparation of standard solutions

Stock solutions of thymol (3 mg/ml) and carvacrol (0.3 mg/ml) were prepared separately in ACN:H_2_O (80:20) solvent. Different concentrations (15–90 µg/ml for thymol and 2–9 µg/ml for carvacrol) were made from stock solutions to plot the calibration curves of thymol and carvacrol.

### Validation

The reliability of HPLC method for analysis of thymol and carvacrol was established through its linearity, selectivity, precision and recovery.

### Linearity

Due to verification of the normal distribution of the results, linearity was evaluated through the relationship between the concentration of thymol and carvacrol and the absorbance obtained from UV-HPLC detector. The determination coefficient (*r*^2^ ) was calculated by means of the leastsquares analysis. The calibration line was achieved through two replicates of each concentration of thymol or carvacrol to know the extent of the total variability of the response that could be explained by the linear regression model.

### Limit of detection and limit of quantization

Limits of detection (LODs) and limits of quantization (LOQs) were calculated using the expressions 3.3σ/*s* and 10σ/*s*, respectively, in which σ is intercept standard deviation and *s* is the slope of calibration curve.[14,15]

### Selectivity

For chromatographic method, developing a separation involves demonstrating specificity which is the ability of the method to accurately measure the analyte response in the presence of all interferences. So, the solutions obtained from sample preparation were analyzed and the analytes’ peaks (thymol and carvacrol) were evaluated for peak purity and resolution from the nearest eluting peak.

### Precision

The precision of each method indicates the degree of dispersion within a series of determinations of the same sample. Three samples in three levels (80, 100, 120%) were analyzed on the same day (intra-day) and for three consecutive days (inter-day) and the relative standard deviations (RSD%) were calculated. Three samples for each level were prepared and each one was injected to HPLC three times.

### Recovery

This parameter shows the proximity between the experimental values and the real ones. It ensures that no loss or uptake occurred during the process. The determination of this parameter was performed for the method by studying the recovery after a standard addition procedure with two additional levels. The concentrations of standards added to the samples were 20 and 50 µg/ml for thymol and 2 and 5 µg/ml for carvacrol. In each additional level, three determinations were carried out and the recovery percentage was calculated in every case. Each sample was injected to HPLC three times.

## Gas chromatographic quantization of thymol and carvacrol

### Sample preparation

Seventy-five milligrams of essential oil was added to a volumetric flask and diluted to 5 ml with hexane. Six samples were prepared and each one was injected to GC instrument three times.

### Preparation of standard solutions

Stock solutions of thymol (20 mg/ml) and carvacrol (2 mg/ml) were prepared in hexane and serial dilutions (2–10 mg/ml for thymol and 0.2–1 mg/ml for carvacrol) were made using stock solutions. The internal standard used was 4-isopropylphenol.

### Statistical analysis

Data were reported as mean ± SD. The results were analyzed statistically by SPSS software and the Student’s *t*-test with level of significance set at *P* < 0.05.

## RESULTS AND DISCUSSION

GC is a common method for the assay of thymol and carvacrol in essential oils and many studies have reported this technique for the quantization of above-mentioned compounds.[1,7,8] But it has some limitations to be used for complicated samples; therefore, this research has been focused on the separation and quantization of thymol and carvacrol by validated HPLC method in *T. vulgaris* essential oil and the results of HPLC method were compared with those of GC technique by statistical analysis.

Thymol and carvacrol peaks were observed in retention times 29.4 and 29.8 min in the GC chromatogram of the essential oil and were well separated. Quantization results showed 40.7 ± 0.6% thymol and 4.2 ± 0.1% carvacrol in *T. vulgaris* L. essential oil [Table 1].

**Table 1:** Comparison of HPLC and GC methods for analysis of thymol and carvacrol in *T. vulgaris* essential oil

Compound	Method			Concentration (%w/w)				Mean ± SD
Thymol	HPLC	41.9	40.8	41.1	41.2	41.3	40.8	41.2 ± 0.4
	GC	40.3	41.6	40.6	39.9	40.9	40.9	40.7 ± 0.6
Carvacrol	HPLC	4.2	4.3	4.4	4.3	4.2	4.2	4.3 ± 0.1
	GC	4.3	4.1	4.2	4.3	4.2	4.1	4.2 ± 0.1

**Figure 2:**
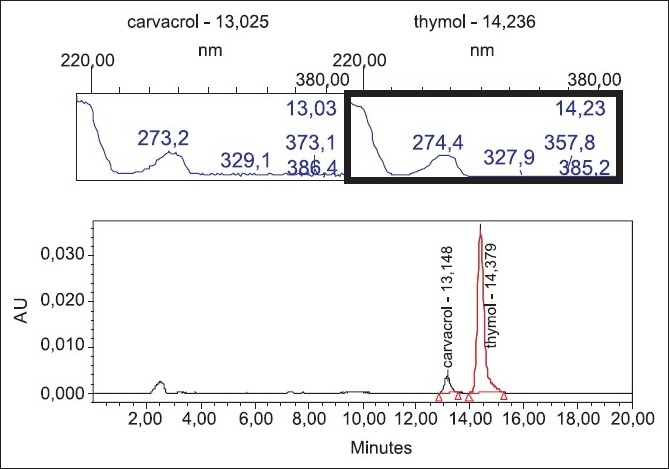
HPLC chromatogram of *T. vulgaris* essential oil with characteristic UV absorption spectra at 200–400 nm

The results of quantitative determination of the two main components of the essential oil by HPLC method demonstrated that thymol and carvacrol peaks were well resolved from each other and displayed excellent peak symmetry and separation efficiency as seen in Figure 2. By using HPLC method, the concentrations of thymol and carvacrol in the essential oil were calculated to be 41.2 ± 0.4 and 4.3 ± 0.1%, respectively [Table 1]. The results obtained from GC and HPLC techniques showed good agreement between two methods (*P* > 0.05).

### Method development

The mobile phase was chosen after several trials with water, methanol and ACN. Finally, a mobile phase consisting ACN:H_2_O (50:50) in an isocratic mode was selected to achieve maximum separation. Flow rates were arranged between 0.5 and 1.5 ml/min. A flow rate of 1 ml/min gave an optimum signal/noise ratio with a reasonable separation time (15 min). After comparison between different columns such as C_8_, C_18_ and CN, the best separation efficiency was obtained using C_18_ column. The retention times for carvacrol and thymol were observed to be 13.1 and 14.4 min, respectively. UV spectrum of thymol and carvacrol showed maximum absorption at 274 nm; therefore, the compounds were monitored at this wavelength using photodiode array detector [Figure 2].

In a similar investigation on thymol and carvacrol in *Molsa chinensis*, ODS column was used with methanol:water:acetic acid (60:40:2) as the mobile phase. The method was validated according to standard validation parameters and it was found to be reliable.[12] This proposed method was tried for analysis of thymol and carvacrol in *T. vulgaris* essential oil but it was not efficient for the separation of two isomers in the above-mentioned plant.

Essential oils contain many components which are usually dissolved in non-polar solvents.[16] In order to achieve best separation of components in HPLC method, the solvent of the sample and mobile phase should be similar. Since the *T. vulgaris* essential oil was not soluble in ACN:H_2_O (50:50), different proportions of ACN:H_2_O were tried and finally 80:20 of ACN:H_2_O was selected for dissolving the sample. In a study performed by Solinas and Gessa for analyzing thymol and carvacrol in *Thymus capitatus* by HPLC, all the samples were prepared in methanol which is not similar to the mobile phase (ACN:H_2_O). This is a probable reason for overlapping of thymol and carvacrol peaks in the chromatogram of the essential oil.[17]

### Validation procedure

The results obtained from method validation for thymol and carvacrol assay according to linearity, selectivity, precision and accuracy showed that the proposed method was reliable [Table 2]. Excellent linearity was obtained for thymol and carvacrol between peak areas and concentrations 15–90 µg/ml with *r*^2^ = 0.9992 for thymol and 2–9 µg/ml with *r*^2^ = 0.9979 for carvacrol. Thymol and carvacrol peaks in the sample and the standard chromatographs were spectrally similar and pure. The three-dimensional and contour plot views of the chromatograms also confirmed the complete separation. LODs and LOQs were calculated as 2.8, 0.6 µg/ml and 8.6, 1.8 µg/ml for thymol and carvacrol, respectively. The precision results for analysis of thymol and carvacrol showed RSD% ≤ 1.9 and 2.6 for intra-day and RSD% ≤ 4.5 and 4.7 for inter-day precision for thymol and carvacrol, respectively, which are reasonable. Accuracy, which was evaluated as recovery after spiking the essential oil with standards at two levels, was found to be 97.7 and 97.6% for thymol and carvacrol, respectively [Table 3].

**Table 2:** HPLC method validation factors for quantization of thymol and carvacrol in *T. vulgaris* L. essential oil

Parameters	Results	
	Thymol	Carvacrol
LOD & LOQ (µg/ml)	2.8 and 8.6	0.6 and 1.8
Selectivity	Selective	Selective
Linearity (*r*^2^)	0.9992	0.9979
Range (µg/ml) Intra-day precision 80, 100, 120% (*n* = 3, RSD%)	15–90 0.8, 1.1, 1.9	2–9 1.9, 1.7, 2.6
Inter-day precision 80, 100, 120%(*n*= 3, RSD%)	3.5, 4.5, 3.6	3.8, 4.7, 3.6

**Table 3:** Recovery study of thymol and carvacrol in *T. vulgaris* L. essential oil by HPLC method

	Concentration in sample (µg/ml)*	Added (µg/ml)	Found (µg/ml)*	Recovery (%)	Mean of Recovery%
Thymol	40.4 ± 0.8	20	59.6 ± 0.6	98.7 ± 1.6	97.7 ± 2.0
		50	87.4 ± 1.2	96.7 ± 1.7	
Carvacrol	4.3 ± 0.05	2	6.2 ± 0.03	98.4 ± 0.7	97.6 ± 1.4
		5	9.0 ± 0.1	96.8 ± 1.5	

## CONCLUSION

This newly established method was validated to be selective, precise and accurate for the quantitative analysis of thymol and carvacrol, the most active components in *T. vulgaris*. It is concluded that this method is not only a useful tool for the assay of these components in *T. vulgaris* essential oil but also an effective quality control method to assay thymol and carvacrol in *Thymus* pharmaceutical dosage forms which could not be analyzed by GC. This method can be considered as a good alternative to the already existing methods for the analysis of these compounds in plants.
